# Intrinsic subthreshold oscillations extend the influence of inhibitory synaptic inputs on cortical pyramidal neurons

**DOI:** 10.1111/j.1460-9568.2010.07146.x

**Published:** 2010-03

**Authors:** Klaus M Stiefel, Jean-Marc Fellous, Peter J Thomas, Terrence J Sejnowski

**Affiliations:** 1 Howard Hughes Medical Institute, Computational Neurobiology Laboratory, The Salk Institute for Biological StudiesLa Jolla, CA, USA; 2 Theoretical and Experimental Neurobiology Unit, Okinawa Institute of Science and TechnologyOkinawa, Japan; 3 Department of Psychology and Applied Mathematics, University of ArizonaTucson, AZ, USA; 4 Departments of Mathematics, Biology, and Cognitive Science, Case Western Reserve UniversityCleveland, OH, USA; 5 Department of Neuroscience, Oberlin CollegeOberlin, OH, USA; 6 Division of Biological Sciences, University of CaliforniaSan Diego, La Jolla, CA, USA

**Keywords:** cortex, Hilbert transform, inhibitory postsynaptic potential (IPSP), oscillations, phase shift

## Abstract

Fast inhibitory synaptic inputs, which cause conductance changes that typically last for 10–100 ms, participate in the generation and maintenance of cortical rhythms. We show here that these fast events can have influences that outlast the duration of the synaptic potentials by interacting with subthreshold membrane potential oscillations. Inhibitory postsynaptic potentials (IPSPs) in cortical neurons *in vitro* shifted the oscillatory phase for several seconds. The phase shift caused by two IPSPs or two current pulses summed non-linearly. Cholinergic neuromodulation increased the power of the oscillations and decreased the magnitude of the phase shifts. These results show that the intrinsic conductances of cortical pyramidal neurons can carry information about inhibitory inputs and can extend the integration window for synaptic input.

## Introduction

Spontaneous membrane potential oscillations are observed in cortical layer 2/3 pyramidal neurons. Oscillations are repeated periodic changes of the membrane potential (Izhikevich, 2006). Subthreshold oscillations of the membrane potential, which constitute the focus of this study, occur without spikes. The oscillations that we investigated are generated intrinsically by the interplay of ionic conductances in the neuronal membrane, rather than being forced by an external source (such as the surrounding neural network). These oscillations occur in the voltage range just below threshold, and are caused by the interaction of a persistent sodium current and at least one slower potassium current. They are typically about 5 mV in amplitude, 4–15 Hz in frequency, and have a stochastic component (Klink & Alonso, 1993, 1997; White *et al.*, 1998; Fellous *et al.*, 2001).

The most prominent effects of synapses on the postsynaptic neurons are postsynaptic changes in the membrane potential, which can be excitatory [excitatory postsynaptic potentials (EPSPs)] or inhibitory [inhibitory postsynaptic potentials (IPSPs)]. The effects of synaptic inputs have been traditionally studied in conditions where the membrane potential was constant and non-fluctuating (Thompson *et al.*, 1990; Magee, 2000; Tamás *et al.*, 2002; Poirazi *et al.*, 2003). However, *in vivo* synaptic transmission is likely to occur under non-stationary conditions, such as ongoing oscillations. One study that addressed the summation of postsynaptic potentials found that EPSP summation during trains of EPSPs and after episodes of action potential firing were not affected by non-stationary conditions (Cash & Yuste, 1999). The effect of subthreshold oscillations on the summation of synaptic potentials in fluctuating membrane conditions has not yet been examined.

The role of GABAergic inhibition in the generation and maintenance of cortical rhythms is now well established (Hasenstaub *et al.*, 2005; Sejnowski & Paulsen, 2006; Mann & Paulsen, 2007), but the influence of single IPSPs on ongoing spontaneous oscillations is still unclear. We examined the mutual influence of IPSPs and intrinsic subthreshold oscillations in pyramidal neurons in cortical slices from rodent occipital cortex *in vitro*, and found strong interactions between the IPSPs and intrinsic subthreshold oscillations.

We also examined the effects of acetylcholine (ACh), a neuromodulator, on the interaction of subthreshold oscillations and IPSPs. An increase in ACh level in the cortex shifts the power in the electroencephalographic oscillations from low frequencies to the γ-frequency band (Steriade, 2004). ACh also influences a number of potassium currents (Madison *et al.*, 1987; McCormick, 1989; Klink & Alonso, 1997), some of which may be involved in the generation of the oscillations under investigation.

## Materials and methods

### Electrophysiology

We recorded from 37 layer 2/3 pyramidal neurons in rat and mouse cortical slices. Animals were treated in accordance with the guidelines of the Salk Institute Animal Care and Use Committee. Rats [Wistar (Harlan, San Diego, CA, USA); postnatal day (P) 18 to P30] or mice [B6D21/Hsd B6, ‘black 6’ (Harlan), P28 to P35] were anesthetized with halothane and decapitated. The occipital forebrain was removed and glued to a plastic block. Coronal slices of the cortex (300 μm) were cut with a Series 1000 Vibratome (Pelco) in ice-cold artificial cerebrospinal fluid (ACSF), containing 125 mm NaCl, 2.5 mm KCl, 1.25 mm NaH_2_PO_4_, 25 mm NaHCO_3_, 2 mm CaCl_2_, 1.3 mm MgCl_2_, and 10 mm dextrose). Slices were allowed to recover in ACSF at 35°C for at least 30 min before the start of recordings. There were no discernible differences in the results between the rat and mouse preparations, so all data were pooled.

Recordings were performed under infrared differential interference contrast video-microscopy in oxygenated ACSF (flow rate, 3 mL/min) at 32°C. Whole-cell patch clamp recordings were performed with electrodes with resistances ranging from 6 to 8 MΩ. The pipette solution contained 140 mm KMeSO_4_, 10 mm Hepes, 1.5 mm NaCl, and 0.1 mm EGTA.

The voltage signal was recorded with an Axoclamp-2A amplifier (Axon Instruments, Foster City, CA, USA), low pass filtered at 30 kHz, and digitized at 10 or 32 kHz with a PCI-MIO-16E-4 DAQ board (National Instruments, Austin, TX, USA). Data acquisition software was custom written in LabVIEW 6.1 (National Instruments).

Glutamatergic ionotropic synaptic transmission was blocked with 6,7-dinitroquinoxaline-2,3-dione (10 μm) in all cases, and (2R)-amino-5-phosphonovaleric acid (50 μm) was added in the majority of cases. In a subset of experiments, GABA_B_ transmission was blocked with saclofen (10 μm). No differences were seen between these experiments, and the data were pooled. Drugs were purchased from Sigma (Dallas, TX, USA) and Fischer (Pittsburgh, PA, USA). GABAergic synaptic transmission was stimulated with an extracellular monopolar glass stimulation electrode, filled with oxygenated ACSF, positioned up to 200 μm away laterally from the recorded neuron in cortical layer 2/3. In other experiments, a negative current pulse (I-pulse) was directly injected at the soma of the cell recorded.

### Data analysis

**Fig1:**
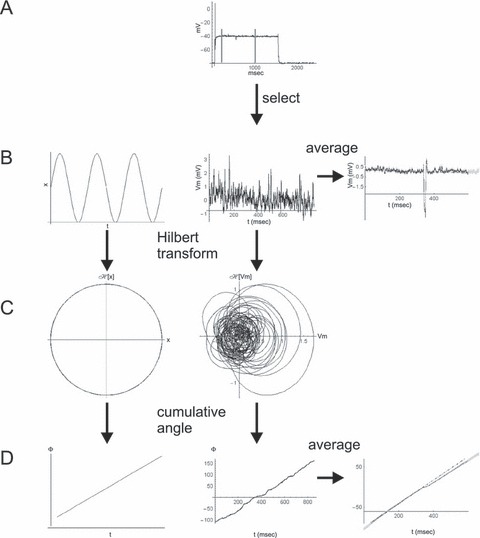
Analysis of phase shifts. Left column: sinusoidal example. Middle column: experimental data. Right column: averages of individual data traces. (A) Raw data. The subthreshold part of the voltage trace before and after the pulse is selected. (B) Data after the average response to the injected current pulse is subtracted and after bandpass filtering. (C) Plot of B against its Hilbert transform. (D) The cumulative angle of the trajectory in C, representing the cumulative phase of the oscillation.

The phase of the oscillation of the membrane potential as a function of time was estimated with the Hilbert transformation, a mathematical method that is tailored for this purpose (see Appendix). Before applying the Hilbert transformation, the data were pre-processed in order to avoid artefacts. First, the voltage signal was bandpass filtered (Fig. 1A) to remove high-frequency noise; then, a β-function was fitted to the IPSP

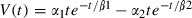
1
or the voltage response to the I-pulse; Fig. 1B, middle graph was subtracted from it to remove the low-frequency signals. These signals were removed to prevent them from introducing artefacts into the Hilbert transformation. The subtraction of the IPSP waveform was performed to improve the precision of our data analysis, and was hypothesis-neutral with respect to the effect of the IPSPs.

After this pre-processing, we plotted the voltage waveform against its Hilbert transform (Fig. 1C, middle graph). The cumulative angle (*Φ*) in this plot represents the cumulative phase of the oscillation, and was plotted as a function of time (Fig. 1D, middle graph). To determine the phase progression of the voltage signal containing the IPSP or I-pulse, we subtracted the averaged phase during 300 ms before the stimulation from the phase after the stimulation (Δ*Φ*; Fig. 1D, right panel). Phase shifts > 2*π* indicated the skipping of more than a complete cycle.

As the stimuli (IPSPs or current injections) are at a fixed time relative to the onset of the depolarization (and hence the oscillations), we did not cover the complete phase (0–2*π*) and were not able to investigate the phase dependence of phase shifts.

We averaged all individual plots of the change in phase, Δ*Φ*, as a function of time of every cell (temporally aligned at the beginning of the IPSP/I-pulse). Then we extrapolated a linear fit to the 300 ms before the IPSP/I-pulse to measure how long it took for the phase signal [± standard error of the mean (SEM); gray area in the plots depicting phases] to cross that extrapolation. We defined the duration of the significant phase shift as the time to the intersection. In the case of deterministic oscillations, a phase shift will persist forever, but in the presence of noise the phase difference will diffuse over time. Eventually, the phase distributions with and without inhibitory perturbation will be indistinguishable. As long as the extrapolation of the baseline is at least one SEM away from the actual phase, they can be reliably distinguished. This measure takes the amount of noise into account, as a phase shift of equal amplitude will diffuse faster (intersect with the baseline extrapolation ± SEM earlier) in the high-noise case.

Also, although alternative definitions of ‘baseline’ are possible, we believe that our choice is reasonable in the context of the questions that we asked. An interval of 300 ms is in the time scales of the processes under investigation, and the linear extrapolation represents the most natural null hypothesis (see below.)

Neurons in which the phase did not return to baseline in less than 250 ms were designated as neurons with a phase shift in response to IPSPs/current pulses. The phase shift (in radians) relative to the linear projection was also determined at 250 ms. When this measure was averaged, only data from neurons in which the phase shift duration exceeded 250 ms were included.

The application of the Hilbert transform-based method to determine the instantaneous phase proved to be remarkably robust against noise and the presence of additional weaker signals in the data. It reliably determined the progression of the cumulative phase of the signal with the most power. A demonstration of this analysis (Hilbert.hoc) running in NEURON (Carnevale & Hines, 2006) is provided as Supporting information, Fig. S1.

We also measured the amount and duration of the phase shifts and the voltage deflections caused by IPSPs and I-pulses, averaged all individual plots of membrane potential vs. time of every cell (temporally aligned at the beginning of the IPSP/I-pulse), and equally extrapolated a linear fit to the 300 ms before the IPSP/I-pulse. We then systematically compared the time that it took for the phase and voltage signals to return to baseline, defined as the intersection (± SEM) with the extrapolation. The significance of these effects across cells was assessed with Student’s *t*-test. Significance tests were always conducted across all neurons of a population (not just the neurons in which an effect was present). These tests were performed over the averages over many sweeps per neuron (22–181) and not the individual sweeps; therefore, they represent a lower bound of the significance of the effects, as testing all sweeps against each other would have yielded higher significances.

## Results

Patch clamp recordings of rat and mouse cortical neurons were performed *in vitro* as described in Materials and methods. All pyramidal neurons used in this study showed pronounced intrinsic subthreshold oscillations in response to a sustained depolarization close to firing threshold.

These oscillatory episodes occurred following current injection, and were preceded by 0–10 action potentials. The presence or number of spikes did not influence the occurrence of oscillations, which were dependent on a sudden depolarization to voltage levels close to firing threshold.

The voltage trace and power spectra of a pyramidal cell are shown in Fig. 2A. Oscillatory frequencies were around 5 Hz (Fig. 1A). Both the frequency and the amplitude of oscillations were non-stationary, most likely owing to the stochastic processes involved in their generation (White *et al.*, 1998). In all neurons, the amplitude of the oscillation was damped (amplitude decreased over time). These were features of the neural process that we were interested in studying.

**2:**
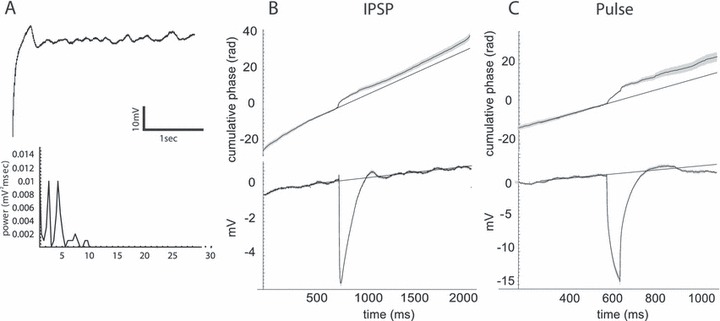
Properties of the subthreshold oscillations. (A) Voltage response to a current pulse depolarizing the neuron just below firing threshold (top) and power spectra averaged over eight voltage sweeps (bottom). (B) Phase shifts caused by inhibitory postsynaptic potentials (IPSPs). (C) Phase shifts caused by current pulses. In B and C, each trace is the mean ± standard error of the mean over all sweeps in one cell, and the cumulative phase of the subthreshold oscillation (top) and voltage (bottom) are shown.

The observed oscillations are most likely damped, noise-driven oscillations, resulting from a spiral sink-like relaxation towards the resting membrane potential. We believe this to be the case, as similar oscillations occur in many models of spiking [if they have type II excitability (Izhikevich, 2006; Rotstein *et al.*, 2006)]. In such deterministic models, these oscillations occur after spikes or depolarizing pulses, and they then dampen out to zero. The addition of noise maintains the oscillations by continuously perturbing the voltage away from the resting potential (the stable fixed point). However, we are not aware of any time-series analysis methods that can distinguish this case from alternatives (such as a limit cycle) and can therefore not draw definite conclusions about the mechanisms giving rise to the observed oscillation. Nevertheless, this uncertainty does not affect the validity of the analysis performed in this study, as we analyse the phase of the oscillations (independently of the presence of a limit cycle) with the Hilbert transform-based method.

When an extracellularly evoked IPSP was elicited during the oscillations, the phase of the oscillations shifted reliably. We observed a phase shift (lasting > 250 ms) in 10 of 16 pyramidal neurons. The average phase shift measured 250 ms after the IPSP was 3.3 ± 0.46 rad (average of 51 sweeps per neuron). Owing to the inherently high noise levels in the voltage and hence the phase, it was not possible to measure a smooth curve describing the phase shift as a function of the phase (a phase reset curve).

In five of the 10 neurons, the phase shift persisted until the end of the sweep (average of 1719 ms, *n* = 5). In the remaining five neurons, the significant shift, defined as the time before the phase intersected with a linear extrapolation of the pre-stimulus phase ± SEM (the gray area in the figures depicting phases; see Materials and methods), terminated, on average, 725 ms (*n* = 5) after the IPSP. The phase shift thus persisted, on average, for at least 1.22 s (*n* = 10), far beyond the duration of the stimulating IPSPs.

In contrast to the phase shifts, the voltage signal returned to baseline significantly more rapidly. In eight of the 10 pyramidal neurons that displayed a phase shift in response to an IPSP, the voltage signal returned to baseline 330 ± 123 ms, on average, after the IPSP. In the remaining two cells, the duration could not be measured because of a pre-existing voltage trend that was unrelated to the IPSP. The significant phase shifts thus lasted 3.7 times longer in pyramidal neurons (*P* < 0.0051) than the IPSPs. This indicated that phase shifts of the membrane potential oscillation retained information about past inhibitory synaptic events for durations that were much longer than the duration of the events themselves measured as voltage shifts.

In a subset of neurons, the oscillations appeared in the averaged voltage traces before the onset of the IPSP (six of 13 neurons; Fig. 2B, bottom traces). This occurred because the onset of the depolarizing pulse (or the action potentials elicited by the pulse) reset the oscillation to an identical initial phase, and the frequency of the oscillation was sufficiently stationary so that they remained phase-locked up to the time when the IPSP was elicited. The action potentials served as a reset for the oscillations under investigation.

In another subset of cells (two of 13 neurons), oscillations appeared in the before the onset of the IPSP and in the averaged voltage traces after the IPSP (Fig. 2B, bottom traces). In these cells, the IPSPs performed phase resetting. The phase shift was not correlated with the standard deviation of the voltage during the oscillations or with the power of the oscillations in the 1–5-Hz and 5–15-Hz bands (not shown).

Next, we determined whether a synaptic conductance change is necessary to evoke a phase shift, or whether a voltage deflection alone also suffices. For that purpose, we injected short (20–60-ms) hyperpolarizing current pulses (I-pulses, −10 to −60 pA) during oscillatory episodes. The results of these experiments mirrored those obtained with IPSPs. We observed a phase shift in 24 of 25 pyramidal neurons. The average phase shift measured 250 ms after the pulse was 1.3 ± 0.31 rad (average of 71 sweeps per neuron). In eight neurons, the phase shift persisted until the end of the sweep. The average phase shift lasted for 1.13 ± 0.2 s, 6.47 times the duration of the average length of the voltage deflection (175 ms, *P* = 3.3 × 10^−5^). The significant phase shift outlasted the voltage shift by a ratio of 1.5 or more in 19 of 25 pyramidal neurons.

We also studied the effects of cholinergic modulation on the interaction of IPSPs with these subthreshold oscillations. We investigated the phase shift caused by IPSPs (four neurons) and current pulses (10 neurons) in the presence of 10 or 20 μm carbachol, a cholinergic receptor agonist. The power of the subthreshold oscillations typically increased and contained more high-frequency components (Fig. 3).

**3:**
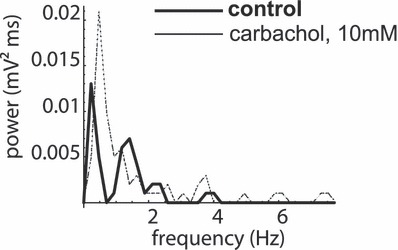
Voltage power spectra averaged over eight voltage sweeps in the absence (thick line) and presence (thin line) of 10 μm carbachol.

In three of four neurons, the IPSPs shifted the phase of the subthreshold oscillation for more than 250 ms. In the neuron where we did not observe an effect in the presence of carbachol, there was also no effect under control conditions. Interestingly, in one neuron, an enduring significant phase shift under control conditions (2400 ms) disappeared when the neuron was exposed to a low concentration of carbachol (10 μm, 77 ms), only to reappear again at a higher concentration of carbachol (20 μm, 896 ms). This is consistent with carbachol having a biphasic effect of cholinergic neuromodulation on cellular excitability (Madison *et al.*, 1987; Stiefel *et al.*, 2009). The average significant phase shift at 250 ms past the IPSP slightly decreased, to 2.96 rad, and outlasted the voltage deflection caused by the inhibitory synaptic input by a factor of 4.39 (1.86-s phase and 425-ms voltage).

Current pulses shifted the phase of the subthreshold oscillation in five of 10 neurons, down from nine of 10 under control conditions (see Fig. 4). The significant phase shift lasted for 1470 ± 268 ms (control conditions), 457 ± 221 ms (10 μm carbachol, *n* = 10) and 1270 ± 678 ms (20 μm carbachol, *n* = 3), also mirroring a biphasic effect of low and high neuromodulatory concentrations. These phase shifts outlasted the pulse’s voltage perturbations by factors of 7.4, 2.5 and 14.09, respectively. The average phase shift caused by current pulses (at 250 ms) was reduced by carbachol to 0.85 ± 0.45 rad (10 μm) and 1.66 ± 0.46 rad (20 μm).

**4:**
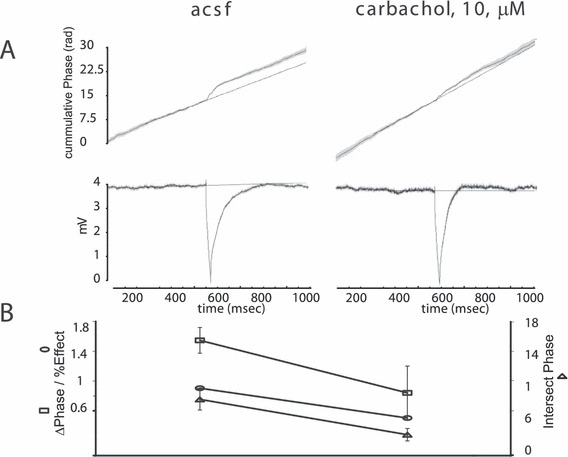
Properties of phase shifts and influence of carbachol. Left column: control condition [artificial cerebrospinal fluid (ACSF)]. Right column: 10 μm carbachol. (A) Example of phase shift evoked by a current pulse in a pyramid (see Fig. 1). (B) Change in the fraction of cells displaying a phase shift in response to the injection of a current pulse (○), ΔPhase 250 ms after the pulse (radians, □) and ratio of the intersection of the phase and voltage with an extrapolation of the trace before the pulse (△). Averages over 10 pyramidal neurons with 85 responses, and 69 sweeps per cell. Error bars represent standard error of the mean.

Because the significant phase shifts induced by IPSPs or current pulses persisted for extended periods of time, phase shifts from a pair of stimuli spaced temporally far apart should summate or interact. To test this hypothesis, we elicited pairs of IPSPs (five intervals in two cells) or current pulses (seven intervals in two cells) 300–800 ms apart (Fig. 5). Whereas voltage deflections spaced more than 200 ms apart returned back to baseline and did not summate, the elicited phase shifts interacted in a subset of experiments.

**5:**
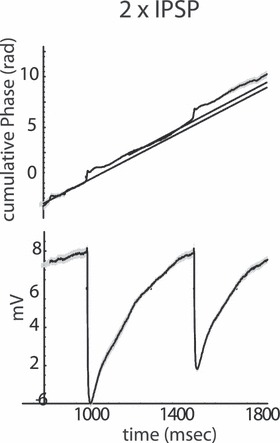
Phase shifts in response to a pair of inhibitory postsynaptic potentials (IPSPs). The two IPSPs (bottom) were separated by 500 ms. The cumulative phase (see Fig. 1) jumped after each IPSP.

In experiments conducted with pairs of IPSPs, the voltage deflections caused by the IPSPs never summed, whereas the phase shift interacted in three of five cases. In two cases (Δ*t* = 300 ms and 800 ms), the phase shifts caused by the first IPSP were positive (+1.9 rad and +2.43 rad), and the phase shifts caused by the second IPSP were negative (−2.6 rad and −2.47 rad). In another case (Δ*t* = 500 ms), both phase shifts were positive (+0.8 rad and +0.9 rad). The phase shifts caused by the second IPSPs persisted until the end of the recordings in all cases. In similar experiments with pairs of current pulses, the voltage deflections caused by the current pulses also never summed. The first pulses caused phase shifts in five of seven cases. Of these, four intersected with the linear extrapolation of the baseline, on average, after 345 ms, before the second pulse was injected. In one case (Δ*t* = 300 ms), the phase shifts caused by the pulses interacted positively (+2.6 rad and +4.2 rad).

These experiments show that effects on the phase of the subthreshold oscillation caused by IPSPs and current pulses can indeed interact, even though these perturbations were separated by 300–800 ms. We observed both positive (phase shifts adding up) and negative (phase shifts offsetting each other) interactions. This heterogeneity is probably explained by additional factors, such as the oscillation frequency, power and phase, that have a non-linear influence on these interactions.

## Discussion

Neurons have a rich repertoire of ionic currents capable of subthreshold oscillations that can store the information about the arrival of an IPSP for periods of time much greater than the duration of the initial perturbation. In contrast, a passive integrator with a fixed firing threshold (as modeled with an integrate-and-fire model neuron, devoid of subthreshold oscillations, for example) would not have such a memory, and would fire solely on the basis of synaptic events that occurred on a time scale of one membrane time constant. As the phase of the oscillation can be influenced by IPSPs for up to at least 1 s, this mechanism can therefore create an extended synaptic integration window. We have observed qualitatively similar effects in cortical interneurons (not shown).

A possible readout for the phase information is the increased likelihood of action potential initiation during the peak and rising phase of voltage oscillations. Shifting the phase of these oscillations will cause a shift in the ability of delayed EPSPs to evoke action potentials. A 5-Hz subthreshold oscillation will have a peak in spiking probability every 200 ms. A shift of the phase of the subthreshold oscillation will shift the time for which an EPSP is most effective at firing the neuron by up to half a cycle, or 100 ms. Thus, the efficacy of an EPSP at a given time can be altered from optimal to minimal by an IPSP that had previously shifted the phase of the subthreshold oscillation by some multiple of 100 ms.

It has been previously proposed that one of the major roles of inhibitory inputs is to determine the precise spike timing of their targets (Lytton & Sejnowski, 1991; Van Vreeswijk *et al.*, 1994; Bevan *et al.*, 2002). The results presented here indicate that they can perform that role on longer than expected time scales.

The shift of the phase of intrinsic subthreshold oscillations described here is different from the phase-resetting curves of suprathreshold oscillations (Reyes & Fetz, 1993; Ermentrout, 1996). These phase-resetting curves describe the phase shifts of intrinsic suprathreshold oscillations and occur in a regular spiking regime. We have shown here that spikes also reset the subthreshold oscillations; therefore, this type of integration takes place only during time intervals between spikes. Thus, action potentials serve both as a neuronal output and as an internal reset mechanism for the synaptic integration based on phase shifts. The phenomenon described here is also different from the entrainment of postsynaptic spiking by IPSPs (Cobb *et al.*, 1995), which is an example of a forced oscillation, not of a phase shift of an intrinsic oscillation caused by an IPSP. Cobb *et al.* (Fig. 2) also described a process similar to the one investigated here, phase shifting of subthreshold oscillations by IPSPs, but did not systematically explore and quantitatively analyse it.

The cholinergic agonist carbachol decreased the phase shift caused by individual current pulse perturbations. This result indicates that, in behaviorally active states, under heightened cholinergic modulation, IPSPs would need to be synchronized (i.e. of compound greater amplitude) to have a long-lasting effect on the oscillatory phase of the postsynaptic target.

A number of conditions have to be met for the phenomenon described here to significantly contribute to neuronal functioning *in vivo*.

First, neurons have to operate in the voltage range in which the intrinsic subthreshold oscillations occur. This is the range just below (< 5 mV) threshold. Intracellular recordings of cortical neurons during active states *in vivo* indicate that this is indeed the case (Destexhe & Paré, 1999).

Second, the effect must persist during high-frequency synaptic input to be of importance *in vivo*. Whether this is the case depends on whether the observed summation of the phase shifts caused by two IPSPs generalizes to more than two. Our results indicate that the phase shifts caused by two IPSPs summate in a non-linear manner. This is plausible, because the oscillation influences the IPSPs at the same time as the IPSPs influence the oscillation. An IPSP would shift the phase, which would alter the effect of the oscillation on future IPSPs, which would in turn alter their effects on the phase of the oscillation. As in any non-linear oscillator, we would expect that the phase effect of a large number of IPSPs would be a complex non-linear accumulation of the effects of the individual IPSPs. Modeling studies could be used to understand the nature of this accumulation.

Third, the firing frequency of neurons must be low enough to avoid frequent resetting of the synaptic integration based on phase shifts (see above). Particularly in states such as deep sleep, when the cortex displays δ-oscillations (0.5–2 Hz), long inter-spike intervals are expected to match the average durations of the integration intervals measured *in vitro*.

We have focused on IPSPs, in part because they have been implicated in generating cortical rhythms, but also because there are strong inhibitory inputs on the somas of cortical pyramidal cells. EPSPs may also influence the phase of subthreshold oscillations, and should be studied as well, but the results may be complicated by the complex synaptic integration in dendritic tree and active dendrite conductances. Nonetheless, this is an important area for future research.

We conclude that the mechanism described here could have important consequences for cortical function *in vivo* during periods with long inter-spike intervals, complementing time-critical suprathreshold processes (Hájos *et al.*, 2004).

## Appendix

### Appendix: Phase determination using the Hilbert transform

The Hilbert transform (Pikovsky *et al.*, 1997), derived from the analysis of complex analytic functions, provides a convenient approximate method for establishing a phase variable for a fluctuating time-dependent signal. Starting from a function *x*(*t*) defined on the real axis, the transform creates a new function *y*(*t*) such that the complex pair *z*(*t*) = [*x*(*t*) + *iy*(*t*)] is analytic in the complex upper half-plane. The complex phase of *z*(*t*), *ϕ*(*t*) = arctan[*y*(*t*)/*x*(*t*)], gives a useful analog of the phase of the variable *x*(*t*). In the case where *x*(*t*) = cos(*ωt*−*δ*), the quantity *ϕ*(*t*)−*ωt* is equal to *δ*, a constant phase shift. In general, the Hilbert transform is only one of many alternative definitions of phase, but is standard for analysis of noisy oscillators (Pikovsky *et al.*, 1997; Müller *et al.*, 2003).

Formally, the Hilbert transform *H*_*x*_(*t*) of a continuous function *x*(*t*) defined for −∞ < *t* < +∞ is the convolution of *x*(*t*) with 1/*t*, i.e.

2

where *PV* stands for the Cauchy principal value of the integral.

For example, if *x*(*t*) = cos(*ωt*) and *y*(*t*) = sin(*ωt*), then *H*_*x*_(*t*) = *y*(*t*) and *H*_*y*_(*t*) = *x*(*t*), regardless of the frequency *ω*. When *x*(*t*) is defined over a finite interval of time, the convolution integral is truncated to give an approximate result.

## Abbreviations

ACh: acetylcholine
ACSF: artificial cerebrospinal fluid
EPSP: excitatory postsynaptic potential
IPSP: inhibitory postsynaptic potential
P: postnatal day
SEM: standard error of the mean

## Supporting Information

Fig. S1 The phase shifts as a function of the phase of the subtreshold oscillation.

Please note: As a service to our authors and readers, this journal provides supporting information supplied by the authors. Such materials are peer-reviewed and may be re-organized for online delivery, but are not copy-edited or typeset by Wiley-Blackwell. Technical support issues arising from supporting information (other than missing files) should be addressed to the authors.

## References

[b1] Bevan MD, Magill PJ, Hallworth NE, Bolam JP, Wilson CJ (2002). Regulation of the timing and pattern of action potential generation in rat subthalamic neurons in vitro by GABA-A IPSPs. J. Neurophysiol.

[b2] Carnevale N, Hines M (2006). The Neuron Book.

[b3] Cash S, Yuste R (1999). Linear summation of excitatory inputs by CA1 pyramidal neurons. Neuron.

[b4] Cobb SR, Buhl EH, Halasy K, Paulsen O, Somogyi P (1995). Synchronization of neuronal activity in hippocampus by individual GABAergic interneurons. Nature.

[b5] Destexhe A, Paré D (1999). Impact of network activity on the integrative properties of neocortical pyramidal neurons in vivo. J. Neurophysiol.

[b6] Ermentrout B (1996). Type I membranes, phase resetting curves, and synchrony. Neural Comput.

[b7] Fellous JM, Houweling AR, Modi RH, Rao RP, Tiesinga PH, Sejnowski TJ (2001). Frequency dependence of spike timing reliability in cortical pyramidal cells and interneurons. J. Neurophysiol.

[b8] Hájos N, Pálhalmi J, Mann EO, Németh B, Paulsen O (2004). Spike timing of distinct types of GABAergic interneuron during hippocampal gamma oscillations in vitro. J. Neurosci.

[b9] Hasenstaub A, Shu Y, Haider B, Kraushaar U, Duque A (2005). Inhibitory postsynaptic potentials carry synchronized frequency information in active cortical networks. Neuron.

[b10] Izhikevich EM (2006). Dynamical Systems in Neuroscience: The Geometry of Excitability and Bursting.

[b11] Klink R, Alonso A (1993). Ionic mechanisms for the subthreshold oscillations and differential electroresponsiveness of medial entorhinal cortex layer II neurons. J. Neurophysiol.

[b12] Klink R, Alonso A (1997). Ionic mechanisms of muscarinic depolarization in entorhinal cortex layer II neurons. J. Neurophysiol.

[b13] Lytton WW, Sejnowski TJ (1991). Simulations of cortical pyramidal neurons synchronized by inhibitory interneurons. J. Neurophysiol.

[b14] Madison DV, Lancaster B, Nicoll RA (1987). Voltage clamp analysis of cholinergic action in the hippocampus. J. Neurosci.

[b15] Magee JC (2000). Dendritic integration of excitatory synaptic input. Nat. Rev. Neurosci.

[b16] Mann EO, Paulsen O (2007). Role of GABAergic inhibition in hippocampal network oscillations. Trends Neurosci.

[b17] McCormick DA (1989). Cholinergic and noradrenergic modulation of thalamocortical processing. Trends Neurosci.

[b18] Müller T, Lauk M, Reinhard M, Hetzel A, Lücking CH, Timmer J (2003). Estimation of delay times in biological systems. Ann. Biomed. Eng.

[b19] Pikovsky AS, Rosnblum MG, Ospinov GV, Kurths J (1997). Phase synchronization of chaotic oscillators by external driving. Physica D.

[b20] Poirazi P, Brannon T, Mel BW (2003). Arithmetic of subthreshold synaptic summation in a model CA1 pyramidal cell. Neuron.

[b21] Reyes AD, Fetz EE (1993). Effects of transient depolarizing potentials on the firing rate of cat neocortical neurons. J. Neurophysiol.

[b22] Rotstein HG, Oppermann T, White JA, Kopell N (2006). The dynamic structure underlying subthreshold oscillatory activity and the onset of spikes in a model of medial entorhinal cortex stellate cells. J. Comput. Neurosci.

[b23] Sejnowski TJ, Paulsen O (2006). Network oscillations: emerging computational principles. J. Neurosci.

[b24] Steriade M (2004). Acetylcholine systems and rhythmic activities during the waking–sleep cycle. Prog. Brain Res.

[b25] Stiefel KM, Gutkin BS, Sejnowski TJ (2009). The effects of cholinergic neuromodulation on neuronal phase-response curves of modeled cortical neurons. J. Comput. Neurosci.

[b26] Tamás G, Szabadics J, Somogyi P (2002). Cell type- and subcellular position-dependent summation of unitary postsynaptic potentials in neocortical neurons. J. Neurosci.

[b27] Thompson SWN, King AE, Woolf CJ (1990). Activity-dependent changes in rat ventral horn neurons in vitro; summation of prolonged afferent evoked postsynaptic depolarizations produce a d-2-amino-5-phosphonovaleric acid sensitive windup. Eur. J. Neurosci.

[b28] Van Vreeswijk C, Abbott LF, Ermentrout GB (1994). When inhibition not excitation synchronizes neural firing. J. Comput. Neurosci.

[b29] White JA, Klink R, Alonso A, Kay AR (1998). Noise from voltage-gated ion channels may influence neuronal dynamics in the entorhinal cortex. J. Neurophysiol.

