# Supplementary material for: Intrinsic subthreshold oscillations extend the influence of inhibitory synaptic inputs on cortical pyramidal neurons
Source: Eur J Neurosci. 2010 Mar;31(6):1019–26. doi: 10.1111/j.1460-9568.2010.07146.x (PMC2862239; doi:10.1111/j.1460-9568.2010.07146.x)
Supplement: Fig. S1 — The phase shifts as a function of the phase of the subtreshold oscillation. [file ejn0031-1019-sd1.doc]

**EJN7146**

**Supporting information**


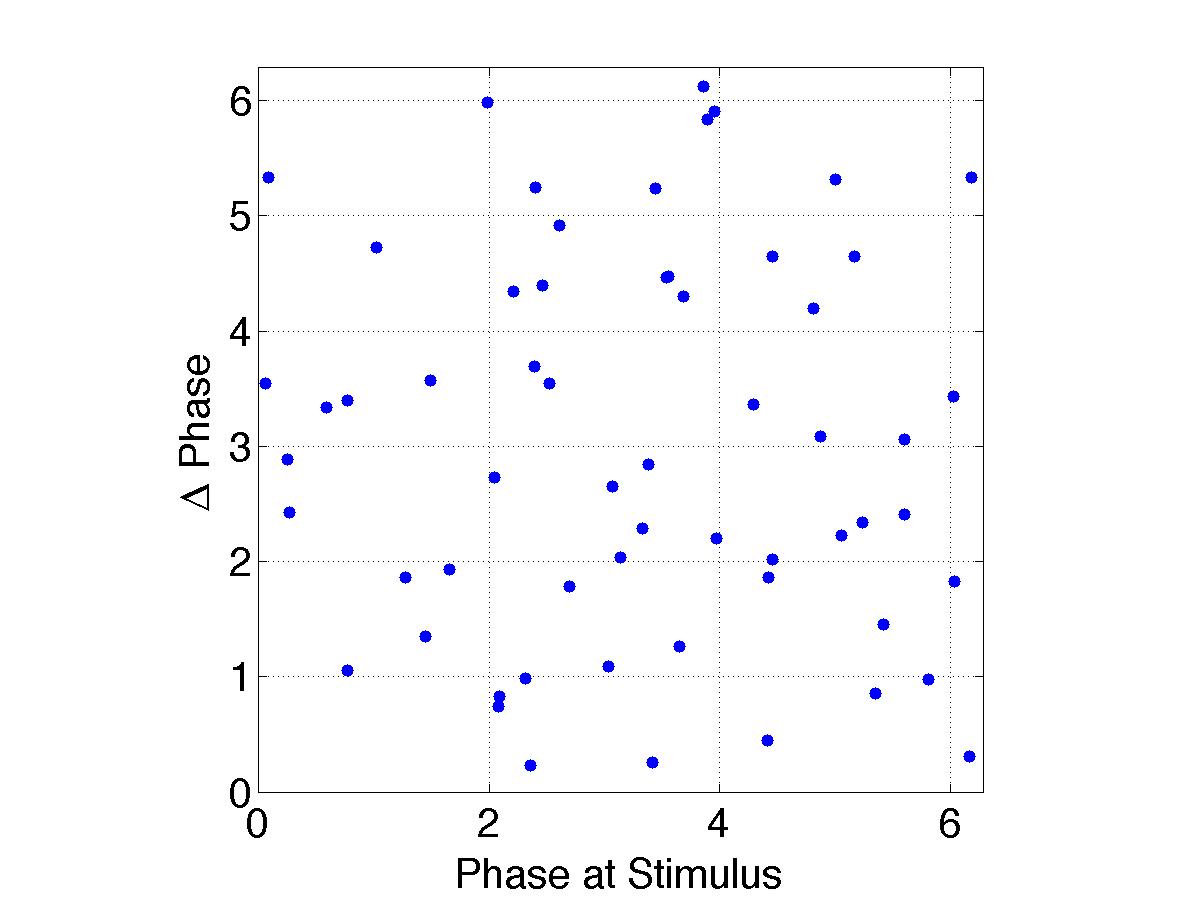


Fig. S1. This figure shows the phase shifts as a function of the phase of the subtreshold oscillation. While there is an overall effect, no clear effect of the phase of the perturbation on the phase shift can be seen.
